# 2,2′-[*p*-Phenyl­enebis(methyl­idene­aza­ne­di­yl)]dipyridinium bis­(hydrogensulfate) dihydrate

**DOI:** 10.1107/S1600536810047999

**Published:** 2010-11-27

**Authors:** Shan Gao, Xiao-Juan Qi, Li-Li Kong, Seik Weng Ng

**Affiliations:** a College of Chemistry and Materials Science, Heilongjiang University, Harbin 150080, People’s Republic of China; b Department of Chemistry, University of Malaya, 50603 Kuala Lumpur, Malaysia

## Abstract

The cation of the title salt, C_18_H_20_N_4_^2+^·2HSO_4_^−^·2H_2_O, lies on a center of inversion with the mid-point directly in the middle of the *p*-phenyl­ene ring. Within the hydrogensulfate ion, the S—O(H) bond is the longest of the S—O bonds. The dihedral angle between the central and terminal ring of the cation is 78.6 (2)°. In the crystal, the cation, anion and water mol­ecule inter­act by O—H⋯O and N—H⋯O hydrogen bonds, generating a three-dimensional network.

## Related literature

For the synthesis and structure of 1,4-bis­(pyridine-2-amino­meth­yl)benzene, see: Zou *et al.* (2003 ▶).
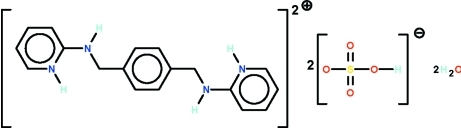


## Experimental

### 

#### Crystal data

C_18_H_20_N_4_^2+^·2HSO_4_^−^·2H_2_O
*M* *_r_* = 522.55
Triclinic, 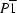

*a* = 7.1718 (5) Å
*b* = 9.3010 (7) Å
*c* = 9.5113 (5) Å
α = 97.648 (2)°
β = 92.340 (2)°
γ = 114.005 (2)°
*V* = 571.25 (7) Å^3^
*Z* = 1
Mo *K*α radiation
μ = 0.30 mm^−1^
*T* = 293 K
0.25 × 0.21 × 0.18 mm

#### Data collection

Rigaku R-AXIS RAPID diffractometer
Absorption correction: multi-scan (*ABSCOR*; Higashi, 1995 ▶) *T*_min_ = 0.930, *T*_max_ = 0.949
5648 measured reflections
2583 independent reflections
1624 reflections with *I* > 2σ(*I*)
*R*_int_ = 0.024

#### Refinement

*R*[*F* ^2^ > 2σ(*F* ^2^)] = 0.040
*wR*(*F* ^2^) = 0.143
*S* = 1.14
2583 reflections
169 parameters
5 restraints
H atoms treated by a mixture of independent and constrained refinement
Δρ_max_ = 0.48 e Å^−3^
Δρ_min_ = −0.42 e Å^−3^

### 

Data collection: *RAPID-AUTO* (Rigaku, 1998 ▶); cell refinement: *RAPID-AUTO*; data reduction: *CrystalStructure* (Rigaku/MSC, 2002) ▶; program(s) used to solve structure: *SHELXS97* (Sheldrick, 2008 ▶); program(s) used to refine structure: *SHELXL97* (Sheldrick, 2008 ▶); molecular graphics: *X-SEED* (Barbour, 2001 ▶); software used to prepare material for publication: *publCIF* (Westrip, 2010 ▶).

## Supplementary Material

Crystal structure: contains datablocks global, I. DOI: 10.1107/S1600536810047999/si2312sup1.cif

Structure factors: contains datablocks I. DOI: 10.1107/S1600536810047999/si2312Isup2.hkl

Additional supplementary materials:  crystallographic information; 3D view; checkCIF report

## Figures and Tables

**Table 1:** Hydrogen-bond geometry (Å, °)

*D*—H⋯*A*	*D*—H	H⋯*A*	*D*⋯*A*	*D*—H⋯*A*
O3—H3⋯O1w^i^	0.84 (1)	1.75 (1)	2.592 (4)	178 (5)
O1w—H11⋯O2	0.84 (1)	1.93 (2)	2.749 (3)	164 (5)
O1w—H12⋯O4^ii^	0.84 (1)	1.98 (1)	2.813 (3)	174 (5)
N2—H2⋯O1	0.89 (1)	2.02 (2)	2.844 (3)	154 (3)
N1—H1⋯O4^iii^	0.88 (1)	2.03 (1)	2.903 (3)	170 (3)

Symmetry codes: (i) 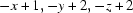; (ii) 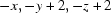; (iii) 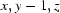.

**Fig. 1.**
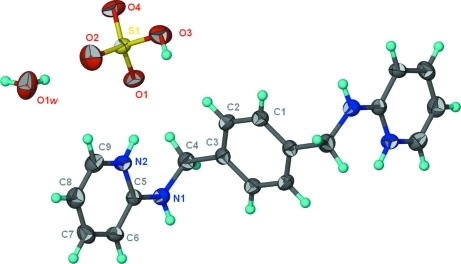
Thermal ellipsoid plot (Barbour, 2001) of C18H20N42+ 2(HSO4)-.2H2O at the 50% probability level; hydrogen atoms are drawn as spheres of arbitrary radius. The cation lies on a center-of-inversion.

## supplementary crystallographic information

### Comment

The synthesis complements other reports of the coordinating ability of 1,4-bis(2-pyridylaminomethyl)benzene, a relatively flexible *N*-donor ligand having aromatic as well as aliphatic donor sites. The structure of the neutral ligand has been reported (Zou *et al.*, 2003). Protonation by a strong acid, sulfuric acid, occurs at both pyridyl nitrogen atoms to yield the hydrogensulfate salt, C_18_H_20_N_4_^2+^ 2(HSO_4_)^-.^2H_2_O (Scheme I, Fig. 1). The cation lies on a center-of-inversion with the mid-point directly in the middle of the *p*-phenylene ring. The hydrogensulfate anion bears a hydrogen atom, so that the S–O_H_ bond is the longest of the S–O bonds. The cation, anion and water molecule interact by O–H···O and N–H···O hydrogen bonds to generate a three-dimensional network (Table 1).

### Experimental

1,4-Bis(2-pyridylaminomethyl)benzene (10 mmol, 2.90 g) was dissolved in methanol (50 ml). Strong sulfuric acid was added until the pH was 3. The solution was filtered; colorless crystals were isolated after several days.

### Refinement

Carbon-bound hydrogen atoms were placed in calculated positions (C–H 0.93–0.97 Å) and were included in the refinement in the riding model approximation, with *U*(H) set to 1.2*U*_eq_(C). The amino/ammonium and water H atoms were located in a difference Fourier map, and were refined with distance restraints of N–H 0.88±0.01 and O–H 0.84 + 0.01 Å; their temperature factors were refined.

### Crystal data

**Table:**

C_18_H_20_N_4_^2+^·2HSO_4_^−^·2H_2_O	*Z* = 1
*M**_r_* = 522.55	*F*(000) = 274
Triclinic, *P*1	*D*_x_ = 1.519 Mg m^−^^3^
Hall symbol: -P 1	Mo *K*α radiation, λ = 0.71073 Å
*a* = 7.1718 (5) Å	Cell parameters from 3948 reflections
*b* = 9.3010 (7) Å	θ = 3.1–27.4°
*c* = 9.5113 (5) Å	µ = 0.30 mm^−^^1^
α = 97.648 (2)°	*T* = 293 K
β = 92.340 (2)°	Prism, colorless
γ = 114.005 (2)°	0.25 × 0.21 × 0.18 mm
*V* = 571.25 (7) Å^3^	

### Data collection

**Table:**

Rigaku R-AXIS RAPID diffractometer	2583 independent reflections
Radiation source: fine-focus sealed tube	1624 reflections with *I* > 2σ(*I*)
graphite	*R*_int_ = 0.024
Detector resolution: 10.000 pixels mm^-1^	θ_max_ = 27.4°, θ_min_ = 3.1°
ω scans	*h* = −9→8
Absorption correction: multi-scan (*ABSCOR*; Higashi, 1995)	*k* = −12→12
*T*_min_ = 0.930, *T*_max_ = 0.949	*l* = −12→12
5648 measured reflections	

### Refinement

**Table:**

Refinement on *F*^2^	Primary atom site location: structure-invariant direct methods
Least-squares matrix: full	Secondary atom site location: difference Fourier map
*R*[*F*^2^ > 2σ(*F*^2^)] = 0.040	Hydrogen site location: inferred from neighbouring sites
*wR*(*F*^2^) = 0.143	H atoms treated by a mixture of independent and constrained refinement
*S* = 1.14	*w* = 1/[σ^2^(*F*_o_^2^) + (0.0586*P*)^2^ + 0.3231*P*] where *P* = (*F*_o_^2^ + 2*F*_c_^2^)/3
2583 reflections	(Δ/σ)_max_ = 0.001
169 parameters	Δρ_max_ = 0.48 e Å^−^^3^
5 restraints	Δρ_min_ = −0.42 e Å^−^^3^

### Fractional atomic coordinates and isotropic or equivalent isotropic displacement parameters (Å^2^)

**Table:**

	*x*	*y*	*z*	*U*_iso_*/*U*_eq_	
S1	0.19334 (11)	0.92763 (8)	0.76081 (7)	0.0379 (2)	
O1	0.1234 (3)	0.7575 (2)	0.7264 (2)	0.0508 (5)	
O2	0.2506 (5)	0.9842 (3)	0.9097 (2)	0.0767 (8)	
O3	0.3889 (4)	1.0040 (3)	0.6846 (3)	0.0627 (7)	
H3	0.481 (5)	0.981 (5)	0.719 (4)	0.094*	
O4	0.0542 (3)	0.9850 (3)	0.7002 (3)	0.0566 (6)	
O1W	0.3282 (4)	1.0635 (4)	1.2016 (3)	0.0730 (8)	
H11	0.286 (7)	1.023 (6)	1.1157 (19)	0.110*	
H12	0.218 (4)	1.055 (6)	1.235 (5)	0.110*	
N1	0.1175 (4)	0.3172 (3)	0.7485 (2)	0.0418 (6)	
H1	0.112 (5)	0.220 (2)	0.740 (4)	0.063*	
N2	0.2258 (4)	0.5652 (3)	0.8961 (2)	0.0388 (5)	
H2	0.202 (5)	0.606 (4)	0.821 (2)	0.058*	
C1	0.5953 (5)	0.6591 (3)	0.4870 (3)	0.0409 (7)	
H1A	0.6588	0.7665	0.4787	0.049*	
C2	0.4067 (5)	0.6003 (3)	0.5401 (3)	0.0417 (7)	
H2A	0.3444	0.6687	0.5668	0.050*	
C3	0.3092 (4)	0.4410 (3)	0.5541 (3)	0.0363 (6)	
C4	0.1038 (5)	0.3747 (4)	0.6143 (3)	0.0432 (7)	
H4A	0.0537	0.4573	0.6301	0.052*	
H4B	0.0057	0.2875	0.5452	0.052*	
C5	0.1854 (4)	0.4078 (3)	0.8764 (3)	0.0342 (6)	
C6	0.2159 (4)	0.3435 (4)	0.9964 (3)	0.0407 (6)	
H6	0.1907	0.2362	0.9874	0.049*	
C7	0.2830 (5)	0.4399 (4)	1.1269 (3)	0.0492 (8)	
H7	0.3020	0.3970	1.2064	0.059*	
C8	0.3230 (5)	0.6011 (4)	1.1422 (3)	0.0502 (8)	
H8	0.3689	0.6664	1.2308	0.060*	
C9	0.2939 (4)	0.6600 (4)	1.0261 (3)	0.0453 (7)	
H9	0.3206	0.7676	1.0345	0.054*	

### Atomic displacement parameters (Å^2^)

**Table:**

	*U*^11^	*U*^22^	*U*^33^	*U*^12^	*U*^13^	*U*^23^
S1	0.0398 (4)	0.0353 (4)	0.0421 (4)	0.0194 (3)	0.0045 (3)	0.0058 (3)
O1	0.0561 (13)	0.0329 (11)	0.0647 (14)	0.0198 (10)	0.0081 (10)	0.0079 (9)
O2	0.106 (2)	0.0816 (19)	0.0359 (13)	0.0403 (17)	−0.0082 (12)	−0.0100 (11)
O3	0.0481 (14)	0.0637 (16)	0.0861 (18)	0.0248 (12)	0.0202 (12)	0.0361 (13)
O4	0.0494 (13)	0.0527 (14)	0.0795 (16)	0.0314 (11)	0.0020 (11)	0.0175 (11)
O1W	0.0499 (15)	0.116 (2)	0.0588 (16)	0.0458 (16)	−0.0023 (12)	−0.0032 (15)
N1	0.0508 (15)	0.0357 (13)	0.0414 (13)	0.0204 (12)	0.0084 (10)	0.0059 (10)
N2	0.0420 (13)	0.0386 (14)	0.0403 (13)	0.0204 (11)	0.0020 (10)	0.0096 (10)
C1	0.0555 (18)	0.0352 (15)	0.0354 (15)	0.0226 (13)	0.0055 (12)	0.0047 (11)
C2	0.0557 (18)	0.0433 (17)	0.0371 (15)	0.0322 (15)	0.0059 (12)	0.0042 (12)
C3	0.0449 (16)	0.0423 (16)	0.0243 (13)	0.0220 (13)	0.0009 (10)	0.0025 (10)
C4	0.0469 (17)	0.0506 (18)	0.0346 (15)	0.0241 (14)	0.0008 (11)	0.0033 (12)
C5	0.0284 (13)	0.0360 (15)	0.0400 (15)	0.0138 (11)	0.0075 (10)	0.0096 (11)
C6	0.0418 (16)	0.0412 (16)	0.0472 (17)	0.0216 (13)	0.0103 (12)	0.0187 (12)
C7	0.0398 (16)	0.073 (2)	0.0443 (17)	0.0278 (15)	0.0096 (12)	0.0258 (15)
C8	0.0475 (18)	0.060 (2)	0.0428 (17)	0.0244 (16)	0.0010 (13)	0.0023 (14)
C9	0.0443 (17)	0.0401 (17)	0.0500 (17)	0.0184 (13)	0.0012 (13)	0.0009 (13)

### Geometric parameters (Å, °)

**Table:**

S1—O2	1.425 (2)	C1—H1A	0.9300
S1—O1	1.437 (2)	C2—C3	1.385 (4)
S1—O4	1.443 (2)	C2—H2A	0.9300
S1—O3	1.551 (2)	C3—C1^i^	1.390 (4)
O3—H3	0.84 (1)	C3—C4	1.517 (4)
O1W—H11	0.84 (1)	C4—H4A	0.9700
O1W—H12	0.84 (1)	C4—H4B	0.9700
N1—C5	1.331 (4)	C5—C6	1.406 (4)
N1—C4	1.462 (4)	C6—C7	1.371 (4)
N1—H1	0.88 (1)	C6—H6	0.9300
N2—C5	1.356 (3)	C7—C8	1.394 (5)
N2—C9	1.362 (4)	C7—H7	0.9300
N2—H2	0.89 (1)	C8—C9	1.341 (4)
C1—C2	1.382 (4)	C8—H8	0.9300
C1—C3^i^	1.390 (4)	C9—H9	0.9300
			
O2—S1—O1	112.24 (15)	C1^i^—C3—C4	119.7 (3)
O2—S1—O4	113.33 (16)	N1—C4—C3	112.3 (2)
O1—S1—O4	113.05 (14)	N1—C4—H4A	109.1
O2—S1—O3	107.05 (17)	C3—C4—H4A	109.1
O1—S1—O3	107.22 (14)	N1—C4—H4B	109.1
O4—S1—O3	103.15 (13)	C3—C4—H4B	109.1
S1—O3—H3	109 (3)	H4A—C4—H4B	107.9
H11—O1W—H12	101 (5)	N1—C5—N2	121.3 (2)
C5—N1—C4	125.7 (2)	N1—C5—C6	121.2 (3)
C5—N1—H1	117 (2)	N2—C5—C6	117.5 (2)
C4—N1—H1	115 (2)	C7—C6—C5	119.6 (3)
C5—N2—C9	122.4 (2)	C7—C6—H6	120.2
C5—N2—H2	118 (2)	C5—C6—H6	120.2
C9—N2—H2	120 (2)	C6—C7—C8	121.0 (3)
C2—C1—C3^i^	120.5 (3)	C6—C7—H7	119.5
C2—C1—H1A	119.7	C8—C7—H7	119.5
C3^i^—C1—H1A	119.7	C9—C8—C7	118.4 (3)
C1—C2—C3	120.9 (3)	C9—C8—H8	120.8
C1—C2—H2A	119.6	C7—C8—H8	120.8
C3—C2—H2A	119.6	C8—C9—N2	121.2 (3)
C2—C3—C1^i^	118.6 (3)	C8—C9—H9	119.4
C2—C3—C4	121.7 (3)	N2—C9—H9	119.4
			
C3^i^—C1—C2—C3	0.3 (5)	C9—N2—C5—N1	179.5 (3)
C1—C2—C3—C1^i^	−0.3 (4)	C9—N2—C5—C6	0.2 (4)
C1—C2—C3—C4	179.0 (2)	N1—C5—C6—C7	−179.0 (3)
C5—N1—C4—C3	80.0 (3)	N2—C5—C6—C7	0.3 (4)
C2—C3—C4—N1	−115.8 (3)	C5—C6—C7—C8	−0.5 (4)
C1^i^—C3—C4—N1	63.5 (3)	C6—C7—C8—C9	0.2 (5)
C4—N1—C5—N2	8.5 (4)	C7—C8—C9—N2	0.3 (5)
C4—N1—C5—C6	−172.2 (3)	C5—N2—C9—C8	−0.5 (4)

Symmetry codes: (i) −*x*+1, −*y*+1, −*z*+1.

### Hydrogen-bond geometry (Å, °)

**Table:**

*D*—H···*A*	*D*—H	H···*A*	*D*···*A*	*D*—H···*A*
O3—H3···O1w^ii^	0.84 (1)	1.75 (1)	2.592 (4)	178 (5)
O1w—H11···O2	0.84 (1)	1.93 (2)	2.749 (3)	164 (5)
O1w—H12···O4^iii^	0.84 (1)	1.98 (1)	2.813 (3)	174 (5)
N2—H2···O1	0.89 (1)	2.02 (2)	2.844 (3)	154 (3)
N1—H1···O4^iv^	0.88 (1)	2.03 (1)	2.903 (3)	170 (3)

Symmetry codes: (ii) −*x*+1, −*y*+2, −*z*+2; (iii) −*x*, −*y*+2, −*z*+2; (iv) *x*, *y*−1, *z*.
